# Real-World Dosing of OnabotulinumtoxinA and IncobotulinumtoxinA for Cervical Dystonia and Blepharospasm: Results from TRUDOSE and TRUDOSE II

**DOI:** 10.3390/toxins13070488

**Published:** 2021-07-14

**Authors:** Ruth Kent, Adrian Robertson, Sandra Quiñones Aguilar, Charalampos Tzoulis, John Maltman

**Affiliations:** 1 Mid Yorkshire Hospitals NHS Trust, Wakefield WF20NJ, UK; adrian.roberson@njs.net; 2 Consultorio de Medicina Especializada del Sector Privado, Mexico City 03100, Mexico; quinonessa@gmail.com; 3 Haukeland University Hospital, University of Bergen, 5021 Bergen, Norway; charalampos.tzoulis@uib.no; 4 Allergan, Irvine, CA 92612, USA; john.matlman@abbvie.com

**Keywords:** botulinum toxins, type A, real-world use, cervical dystonia, blepharospasm

## Abstract

The real-world use of onabotulinumtoxinA and incobotulinumtoxinA for cervical dystonia and blepharospasm treatment was assessed in two separate retrospective studies using identical protocols (TRUDOSE and TRUDOSE II). The studies were conducted in Mexico, Norway, and United Kingdom and designed to evaluate dose utilization of the two botulinum toxins in clinical practice. Eighty-three patients treated with both onabotulinumtoxinA and incobotulinumtoxinA for ≥2 years for each botulinum toxin were included, (52, cervical dystonia; 31, blepharospasm). All patients switched from onabotulinumtoxinA to incobotulinumtoxinA for administrative/financial reasons. A range of dose ratios (incobotulinumtoxinA to onabotulinumtoxinA) was reported; with the majority of dose ratios being >1. The mean dose ratio was >1 regardless of the study site or underlying clinical condition. The inter-injection interval was significantly longer for onabotulinumtoxinA versus incobotulinumtoxinA when assessed for all patients (15.5 vs. 14.3 weeks; *p* = 0.006), resulting in fewer onabotulinumtoxinA treatments over the study time period. Consistent with product labeling, no single fixed-dose ratio exists between incobotulinumtoxinA and onabotulinumtoxinA. The dosage of each should be individualized based on patient needs and used as per product labeling. These real-world utilization data may have pharmacoeconomic implications.

## 1. Introduction

Since 1989, botulinum toxins have gained many approvals for a variety of therapeutic indications worldwide [1,2]. A number of botulinum toxin serotypes exist, with serotype A the most extensively researched and approved for the widest range of therapeutic indications [3,4]. Three botulinum toxin type A products—onabotulinumtoxinA (BOTOX^®^, Allergan plc, Dublin, Ireland), incobotulinumtoxinA (XEOMIN^®^, Merz GmbH & Co, Frankfurt, Germany), and abobotulinumtoxinA (Dysport^®^, Ipsen, Slough, Berks, UK—are commercially available for therapeutic use in Europe, the United States [5], and Mexico and are approved for use in the treatment of cervical dystonia (CD) and blepharospasm. Botulinum toxin type A is considered to be the first-line treatment for both CD [6] and blepharospasm [7].

The availability of multiple botulinum toxins to treat these conditions has led to continued debate regarding potential dose equivalence among products and comparisons in efficacy and safety [4]. There are many factors to consider when comparing botulinum toxin type A products, including differences in clinical performance resulting from underlying differences in manufacturing processes, formulations, and potency. The latter is an important consideration; because each company uses its own proprietary potency assays, differences in assay methodology result in distinct unit potencies and dose-response curves for each product that cannot be compared [8]. Animal studies assessing dose equivalence in vivo confirm that doses of abobotulinumtoxinA, incobotulinumtoxinA, and onabotulinumtoxinA are not interchangeable [8,9].

Nevertheless, comparisons among products and dose equivalence have been investigated in clinical studies with conflicting results. Some noninferiority studies tend to indicate a 1:1 dose ratio on a unit-for-unit basis for incobotulinumtoxinA to onabotulinumtoxinA for the treatment of patients with CD [10,11] and blepharospasm [12,13]. Whereas other studies have reported dose conversions between incobotulinumtoxinA and onabotulinumtoxinA is not a fixed 1:1 dose ratio and/or have differences in median injection intervals [14]. Thus, the use of botulinum toxin type A products should be based on their individual efficacy and safety profiles [4,15].

Randomized clinical trials (RCTs) are the “gold standard” for evaluating therapeutic efficacy and safety; however, by design, they do have strict inclusion/exclusion criteria [16,17]. As a result, RCTs look at a narrow and homogenous population of patients with the evaluated indication under highly controlled clinical conditions. Real-world studies have much broader eligibility criteria that allow for the enrollment of patients that would be seen in everyday clinical practice. The results of these studies can provide necessary information on the clinical utility of drugs in real-world practice that can complement the results of RCTs. Whereas clinical studies comparing the efficacy and safety of onabotulinumtoxinA with the two other botulinum toxin type A products in CD and blepharospasm have been reported [12,18,19,20,21,22], there are limited data comparing the dose utilization of onabotulinumtoxinA and incobotulinumtoxinA in real-world clinical practice.

The primary objective of The Retrospective EvalUation of the DOSE of onabotulinumtoxinA and incobotulinumtoxinA (TRUDOSE) for the Clinical Management of Cervical Dystonia and Blepharospasm studies was to evaluate the real-word dosing of onabotulinumtoxinA and incobotulinumtoxinA for CD and blepharospasm in clinical practice, including the number of and time between injections. These studies were not designed to assess the efficacy of either botulinum toxin and efficacy data was not collected; however, treatment characteristics of each were compared, and the frequency of adverse events (AEs) by toxin and disease type was assessed. These data provide an important contribution to our real-world understanding of the use of botulinum toxin type A in the treatment of CD and blepharospasm.

## 2. Results

### 2.1. Patient Disposition and Demographics

For the TRUDOSE pilot study and TRUDOSE II, 116 and 89 patients, respectively, were screened for eligibility. The most common reason that patients did not meet eligibility criteria was an insufficient period of time of treatment with onabotulinumtoxinA or incobotulinumtoxinA (TRUDOSE pilot, *n* = 62; TRUDOSE II, *n* = 20). Other frequently reported reasons for ineligibility are shown in Table 1.

For the purposes of toxin usage analysis, the evaluable pooled population consisted of 83 patients: 39 enrolled in the TRUDOSE pilot study (Norway, *n* = 11; United Kingdom, *n* = 28) and 44 enrolled in TRUDOSE II (Mexico, *n* = 25; United Kingdom, *n* = 19). The safety analysis set (*n* = 92) included an additional nine patients from the TRUDOSE pilot study. These patients were treated but not included in the toxin usage analysis as they only had 1 year of data before and after the switch.

The majority of patients in both studies had CD. Most patients were female, and the mean age of patients was approximately 65 years (Table 2). More than 90% of patients had either CD or blepharospasm for more than 5 years.

### 2.2. Treatment Characteristics

Pooled data demonstrated numerically lower mean doses per treatment when patients received onabotulinumtoxinA compared with incobotulinumtoxinA, regardless of whether they were receiving treatment for CD (159.1 U vs. 170.7 U, respectively) or blepharospasm (25.9 U vs. 33.4 U). For both studies, a range of dose ratios was observed. The mean dose ratio of incobotulinumtoxinA to onabotulinumtoxinA using the last dose was >1, regardless of the underlying condition (Table 3).

**Figure 1:**
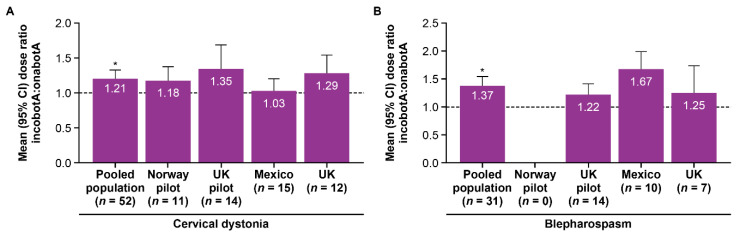
Mean dose ratio of incobotulinumtoxinA to onabotulinumtoxinA (based on last dose) for (**A**) cervical dystonia and (**B**) blepharospasm, stratified by study center. IncobotA = incobotulinumtoxinA; OnabotA = onabotulinumtoxinA. * *p* < 0.0001, one-sample *t*-test where the null hypothesis is dose ratio = 1.0.

Based on the pooled data, the mean dose ratio for incobotulinumtoxinA to onabotulinumtoxinA was significantly >1 for patients with CD (1.21; *p <* 0.0001; Figure 1A), for patients with blepharospasm (1.37; *p <* 0.0001; Figure 1B), and across the entire treatment group (1.27; *p <* 0.0001).

**Figure 2:**
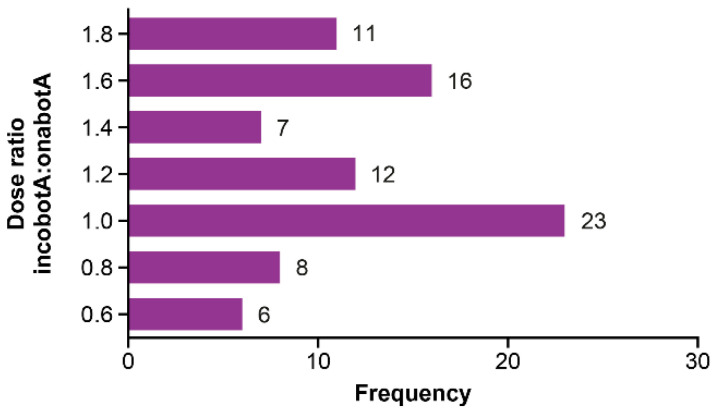
Frequency distribution of the mean dose ratio of incobotulinumtoxinA to onabotulinumtoxinA (based on the last dose) for all patients. IncobotA = incobotulinumtoxinA; OnabotA = onabotulinumtoxinA.

When analyzed by the site, the mean dose ratio was >1 for all sites, both on an overall patient level (range, 1.18–1.28) and when stratified by the underlying condition (CD: range, 1.03–1.35; blepharospasm: range, 1.22–1.67; Figure 1). The dose ratio did not follow a normal distribution (Figure 2). When assessed using the total dose over the 2-year treatment period (incobotulinumtoxinA divided by onabotulinumtoxinA), the mean dose ratio for all patients was significant >1 (1.18; *p <* 0.0001; Table 3) and >1 for all study sites (data not shown).

**Figure 3:**
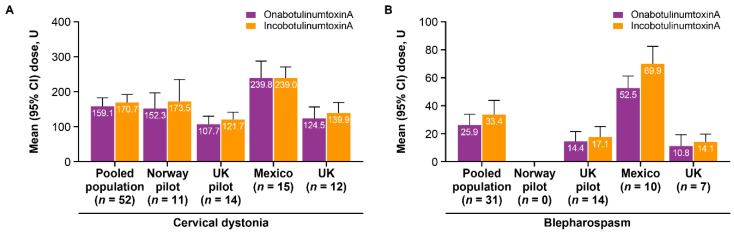
Mean dose per treatment of onabotulinumtoxinA and incobotulinumtoxinA over the duration of the study for (**A**) cervical dystonia and (**B**) blepharospasm, stratified by study center. CI = confidence interval.

Mean dose per treatment for both onabotulinumtoxinA and incobotulinumtoxinA was lower for CD in the TRUDOSE pilot study compared with TRUDOSE II (127.3 U and 144.5 U vs. 188.6 U and 194.9 U, respectively; Table 4); a similar trend was observed for blepharospasm (14.4 U and 17.1 U vs. 35.3 U and 46.8 U). This difference in mean dose was driven by higher doses used at the Mexican study site in TRUDOSE II. At the Mexican site, the mean dose of onabotulinumtoxinA and incobotulinumtoxinA for CD was 239.8 U and 239.0 U, respectively, versus 159.1 U and 170.7 U for all patients with CD; similar trends were observed for blepharospasm (52.5 U and 69.9 U vs. 25.9 U and 33.4 U; Figure 3).

**Figure 4:**
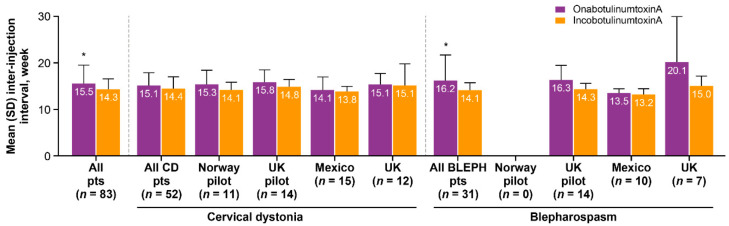
Mean inter-injection intervals for onabotulinumtoxinA and incobotulinumtoxinA stratified by condition and study center. BLEPH = blepharospasm; CD = cervical dystonia; SD = standard deviation. * *p* < 0.05, paired *t*-test (Bonferroni correction for multiple comparisons).

The inter-injection interval between onabotulinumtoxinA treatments and between incobotulinumtoxinA treatments was significantly different across the pooled population (15.5 vs. 14.3 weeks, respectively; *p* = 0.006) and in all patients treated for blepharospasm (16.2 vs. 14.1 weeks; *p* = 0.02; Figure 4).

The mean inter-injection interval was longer for onabotulinumtoxinA than for incobotulinumtoxinA in each of the individual sites. The difference in pooled inter-injection intervals was one or more weeks longer for onabotulinumtoxinA compared with incobotulinumtoxinA.

All patients switched from onabotulinumtoxinA to incobotulinumtoxinA. The reason for the switch was tender/financial (TRUDOSE pilot, *n* = 39; TRUDOSE II, *n* = 19) or due to a change in formulary at the institution (TRUDOSE II, *n* = 25) and not as a result of any efficacy or safety concerns.

### 2.3. Safety and Tolerability

The medical records of 92 patients were reviewed for AEs, including records of 48 patients enrolled in the TRUDOSE pilot study and 44 patients enrolled in TRUDOSE II. AEs occurred in 22 patients (23.9%) in this pooled population, with no serious or severe AEs reported (Table 5).

The type of botulinum toxin type A product received did not appear to have any direct influence on the nature or incidence of AEs. Patients receiving botulinum toxin therapy for blepharospasm were more likely to report an AE (10/34 patients; 29.4%) than patients receiving treatment for CD (12/58 patients; 20.7%). Patients who received treatment for CD most commonly reported AEs of dysphagia (*n* = 6) and head drop (*n* = 4), whereas patients who received treatment for blepharospasm most commonly reported an AE of ptosis (*n* = 7; Table 6).

## 3. Discussion

Patients treated with onabotulinumtoxinA required lower doses than those treated with incobotulinumtoxinA, based on the dose ratio at the end of the 2-year treatment period, regardless of the underlying condition. The mean dose ratio (incobotulinumtoxinA to onabotulinumtoxinA) for the last dose and over the 2-year treatment period was >1 in both studies and in the pooled population. The inter-injection interval between onabotulinumtoxinA treatments and between incobotulinumtoxinA treatments was different across the pooled population and in all patients being treated for blepharospasm.

All study outcomes were largely consistent between the TRUDOSE pilot study and the TRUDOSE II study. However, the higher doses of onabotulinumtoxinA and incobotulinumtoxinA given at the study site in Mexico for both CD and blepharospasm in TRUDOSE II resulted in higher average doses in TRUDOSE II than in the TRUDOSE pilot study for both botulinum toxins. The reasons for the difference in doses in Mexico are unclear but could be due to differences in clinical management (i.e., conservative vs. aggressive management). In addition, therapeutic doses of botulinum toxins may vary depending on the size and number of the target muscles, the degree of dystonia being treated [23], and the degree of pain present [24].

While the potential dose ratio for conversion among the botulinum toxin type A products has been a matter of interest for more than two decades [25], there remains no consensus on a fixed-dose ratio when switching between toxins. Some noninferiority trials have reported similar efficacy outcomes when a 1:1 dose ratio for incobotulinumtoxinA to onabotulinumtoxinA was used to treat patients with CD [10,11] or blepharospasm [12,13,26]. Similarly, switching patients from onabotulinumtoxinA to incobotulinumtoxinA using a 1:1 ratio in a real-life clinical setting did not result in any subjective or objective differences in therapeutic outcomes in patients with a range of conditions, including CD and blepharospasm [27]. In contrast, others have found that a 1:1 ratio results in a trend toward improved efficacy outcomes with onabotulinumtoxinA compared with incobotulinumtoxinA in the treatment of blepharospasm, suggesting that a 1:1 dose ratio does not yield similar outcomes [26]. Furthermore, a large real-world observation of patients (*n* = 288) with blepharospasm reported a conversion ratio for onabotulinumtoxinA versus incobotulinumtoxinA of 1.2 (coefficient variance 0.2), with onabotulinumtoxinA doses 16.7% lower than incobotulinumtoxinA doses [18].

With respect to its potential to treat blepharospasm, studies suggest that onabotulinumtoxinA (20 U) and abobotulinumtoxinA (60 U) significantly outperform incobotulinumtoxinA (20 U) based on dynamic strain reduction after injection into the glabella [28], and sustained improvements in facial synkinesias at week 4 [29].

Botulinum toxins are complex biologic medications that differ in manufacturing processes, formulations, potency units, serotypes, immunogenicity, and quality control factors that have clinical implications [8]. Several preclinical studies have demonstrated differences between onabotulinumtoxinA and incobotulinumtoxinA, which appear to be driven by differences in molecular size, and include the ability to dissociate from complexing proteins and time to death assay in mice [30]. Furthermore, the unit dose potency differs between these two types of botulinum toxin A when using different LD50 assays (i.e., onabotulinumtoxinA > incobotulinumtoxinA in onabotulinumtoxinA LD50 assay, but potency equivalent in incobotulinumtoxinA LD50 assay) [31,32]. Studies by Brown, Kutshenko, and Rupp have also demonstrated in various in vivo and in vitro assays that the potency units of onabotulinumtoxinA and incobotulinumtoxinA are not interchangeable [33,34].

We found that the dose ratio relationship between the two botulinum toxins was much more complex in the real world than was suggested by consensus panels [35] or meta-analyses of clinical trials [36], both of which concluded the botulinum toxins were equipotent and could be switched using a 1:1 conversion ratio. In this study, the incobotulinumtoxinA to onabotulinumtoxinA dose ratio was >1 across both conditions; there was considerable variability in the dose ratio and the inter-injection interval at an individual patient level, but mean dose ratios were all >1 and are consistent with the results of Kollowe and colleagues [18], Wilson [28], Thomas [29], and the preclinical studies cited above. Our results support that there is no fixed-dose ratio conversion between incobotulinumtoxinA and onabotulinumtoxinA, and consistent with the product label and recommendations from regulatory agencies, the potency units of onabotulinumtoxinA are not interchangeable with other botulinum toxin type A products [37]. In accordance with previous recommendations, these results suggest dosing should be adjusted based on individual patient needs and not on a prespecified dose ratio [4,15]. This observation is even more noteworthy in our study, in which patients served as their own controls.

Neither efficacy nor safety concerns were the underlying reason for switching botulinum toxin therapy in either study; administrative reasons (the results of a tender or a change in the formulary at an institute) led to the switch from onabotulinumtoxinA to incobotulinumtoxinA. The direct costs (i.e., clinician time, clinic visits, drug and administration costs) and indirect costs (time off work for additional clinic visits, cost of travel, etc.) should be considered as part of the overall financial implications of any switch.

Given that inter-injection intervals are longer and total doses lower with onabotulinumtoxinA versus incobotulinumtoxinA, one would expect this to translate into lower healthcare-related treatment costs, greater patient convenience, and lower indirect costs (e.g., less absenteeism). For example, when averaging the mean total dose per patient per year in the two studies, on average 111 U per year less onabotulinumtoxinA is used than incobotulinumtoxinA for the treatment of CD and on average 43 U per year less for the treatment of blepharospasm. Our study results could be used to inform a subsequent pharmacoeconomic analysis to enable more informed botulinum toxin selection from patient convenience and economic perspectives.

The longer inter-injection interval reported here for onabotulinumtoxinA is interesting and consistent with the findings of others [18,38]. Indeed, Chundury and colleagues reported that patients who preferred onabotulinumtoxinA over incobotulinumtoxinA most frequently cited the longer treatment duration with onabotulinumtoxinA as the reason for their preference. As this was a retrospective observational study, a variety of factors could have influenced the inter-injection interval, including patient and physician availability, reimbursement protocols, and patient scheduling issues in addition to potential efficacy considerations. Treatment delays observed in other studies, even when treatment was essentially free, included scheduling issues (conflicted with work, vacations, or hospitalization), patient-initiated delays to test treatment duration, condition severity, or a fear of adverse effects, or general compliance issues [39]. Nevertheless, the observation of a longer inter-injection interval for patients treated with onabotulinumtoxinA was generally consistent across sites and indications in the present study.

This study has several limitations. As this was a retrospective, noninterventional study, dosing, muscle localization, injection technique, and dilution were at the full discretion of the treating physician. When switching between botulinum toxins, physicians dosed the patients with the assumption of a 1:1 dose ratio. As this was an international multicenter study, differences in injection technique at an individual, as well as at a country level, may have varied considerably and may have affected the results. Indeed, there were marked differences in the doses used in Mexico compared with those used in the United Kingdom and Scandinavia. Furthermore, this study investigated drug utilization and did not consider efficacy outcomes, although it can be assumed in this real-world setting that physicians were aiming to achieve optimal efficacy for their patients. Despite the differences in treatment approach, all study sites reported a mean dose ratio > 1 when switching from onabotulinumtoxinA to incobotulinumtoxinA, suggesting widespread generalizability of the primary outcome of this study. It has been reported that patients with increased disease severity or the presence of pain may utilize higher doses of onabotulinumtoxinA [40], and the increases in dose seen in this study may have been driven by such changes in the disease state. Over long periods of time, patients may require dose adjustments, including increases in dose; however, treatment intervals typically increase with subsequent treatments [41]. Further, no formal sample size calculation was undertaken to determine if the study was sufficiently powered to detect a difference in the mean dose ratio across the study. Additionally, the patient numbers enrolled at each site and for each condition were relatively small. Nonetheless, the consistency of outcomes across the individual study sites across the 4-year study period suggests that this study was of sufficient size to detect the underlying differences in mean dose ratio. Finally, the switch of botulinum toxin was unidirectional; all patients switched from onabotulinumtoxinA to incobotulinumtoxinA. Further study would be required to assess the effect on dose ratios of switching from incobotulinumtoxinA to onabotulinumtoxinA.

## 4. Conclusions

This study has shown that no single fixed-dose ratio relationship between onabotulinumtoxinA and incobotulinumtoxinA exists and that dosing should be based on individual patient needs, consistent with product labeling. These real-world utilization data and clinical outcomes data may have pharmacoeconomic implications for the use of botulinum toxins in patients with CD or blepharospasm.

## 5. Materials and Methods

### 5.1. Study Design

The TRUDOSE studies were multicenter, noninterventional, retrospective, medical record reviews of the real-world use of onabotulinumtoxinA and incobotulinumtoxinA for the clinical management of CD or blepharospasm.

The TRUDOSE pilot study was conducted at two investigational sites in the United Kingdom and Norway; the TRUDOSE II study, which used the same methodology and was undertaken after completion of the pilot study, was conducted at two investigational sites in the United Kingdom and Mexico. De-identified patient data were abstracted from medical records for eligible patients and were transcribed to case report forms for analysis.

For Mid Yorkshire Hospitals ethical approval was not required as this was an institutional economic switch, patients records were reviewed retrospectively. It was not deemed to fit the criteria of research so ethics was not required for a service review. Identifiable data were not collected; patient consent was not required for these noninterventional studies.

### 5.2. Patients

Patients aged 18 years or older at the date of their first botulinum toxin injection with a confirmed primary diagnosis of either idiopathic CD or blepharospasm were eligible for the study. Additional inclusion criteria were the treatment with either onabotulinumtoxinA or incobotulinumtoxinA for at least 2 years immediately before switching to the alternate botulinum toxin for at least 2 years immediately after the switch (before 30 November 2012, for the TRUDOSE pilot study and before 31 January 2014, for TRUDOSE II).

Patients were excluded from the study if they had any neuromuscular junction transmission disorder or were taking any medication (e.g., pyridostigmine, neostigmine, dantrolene, tubocurarine, streptomycin, aminoglycosides) that could affect neuromuscular junction transmission. Additional exclusion criteria were previous surgical procedures involving bone or muscle for the management of CD or blepharospasm, participation in a clinical trial, change in their adjuvant pharmacotherapy toxin (e.g., clonazepam, anticholinergics) for dystonia that could either affect dystonia severity or result in increased AEs at any time during the study period, and clinical nonresponsiveness to any of the botulinum toxin type A products available at the time of the study—abobotulinumtoxinA, incobotulinumtoxinA, or onabotulinumtoxinA—either before or during the study period.

Injection methodology, including dosing and muscle localization, was left to the discretion of the treating physician. Upon switching between botulinum toxins, patient care was continued at the same institution.

### 5.3. Study Measures

The primary outcome measure was the ratio of the last dose of incobotulinumtoxinA to the last dose of onabotulinumtoxinA in each patient for CD and blepharospasm. A secondary outcome measure assessed dose ratio on the basis of the total dose over each 2-year treatment period before and after the switch. Other treatment-related information was also collected, such as time between treatments, number of treatments, number of units of botulinum toxin used at each treatment for the 2 years immediately before or after the switch, and the reason for switching from one botulinum toxin type A product to the other.

No formal efficacy and safety outcomes were assessed as part of the study; however, AE data were extracted from the medical records to assess the frequency of AEs by toxin and disease type. Safety data included subjective or objective symptoms provided by the patient and/or observed by the investigator or medical staff and documented in the medical chart and physical examination findings. Signs, symptoms, and/or abnormalities existing before the use of onabotulinumtoxinA or incobotulinumtoxinA were not considered AEs unless they recurred during the study period or represented a clinically significant exacerbation in intensity or frequency in the opinion of the study investigator.

### 5.4. Statistical Analyses

Data from the TRUDOSE pilot study and TRUDOSE II were pooled for the key study measures (demographics, dose ratios, and treatment characteristics [e.g., inter-injection intervals]). Patient demographic data were summarized using descriptive statistics; frequencies and percentages were reported for categorical data. Because all patients switched from onabotulinumtoxinA to incobotulinumtoxinA, no stratification or distinction was required.

The total dose over each 2-year treatment period was calculated based on the mean of all incobotulinumtoxinA doses divided by the mean of all onabotulinumtoxinA doses. The mean, standard deviation, and 95% confidence interval (CI) were calculated for the dose ratio and stratified by toxin and disease type. A one-sample *t*-test was used to test whether the dose ratio was equal to 1.0, using the Bonferroni correction for multiple comparisons.

Inter-injection intervals were analyzed by medication and disease type, based on the average number of days between consecutive treatments before and after the switch. An inferential test was performed to assess whether the inter-injection intervals or average annual toxin utilization rates per patient were the same when compared by the toxin. If the distributional assumptions were met, the paired *t*-test was used to test if there was a difference in inter-injection intervals, using the Bonferroni correction for multiple comparisons.

Safety data were summarized by the number of patients experiencing at least one AE by toxin and disease type; the proportion and 95% CI of patients who experienced at least one AE were reported.

All *p*-values were nominal and no sample size calculations were performed for this study.

## Figures and Tables

**Table 1:** The most frequently reported reason for screening failure.

	TRUDOSE Pilot	TRUDOSE II
Patients screened, *n*	116	89
Ineligible patients that did not meet inclusion/exclusion criteria, *n*	77	45
Insufficient treatment time	62	20
Lack of confirmed diagnosis ≥ 2 years before the switch	11	0
Surgical procedure involving bone or muscle for disease management	4	7
Enrollment in another clinical trial	0	7
Changes in adjuvant therapy for dystonia that could affect disease severity or result in increased adverse events	0	7
Evaluable patients, *n*	39	44

**Table 2:** Baseline patient demographics and clinical characteristics for both TRUDOSE studies.

Characteristic	TRUDOSE Pilot (*n* = 39)	TRUDOSE II (*n* = 44)	Pooled Data (*n* = 83)
Mean (SD) age at enrollment, y	63.5 (12.8)	65.4 (11.4)	64.5 (12.1)
Median (range) age, y	65.0 (33.0–88.0)	64.0 (32.0–≥90.0) *	65.0 (32.0–≥90.0) *
Female, *n* (%)	28 (71.8)	32 (72.7)	60 (72.3)
**Condition, *n* (%)**			
Cervical dystonia	25 (64.1)	27 (61.4)	52 (62.7)
Blepharospasm	14 (35.9)	17 (38.6)	31 (37.3)
**Time since onset of condition, *n* (%)**			
>5 years	36 (92.3)	44 (100.0)	80 (96.4)
2–5 years	3 (7.7)	0 (0.0)	3 (3.6)

* Two patients were ≥90 years old; their date of birth was not included in the database. SD = standard deviation.

**Table 3:** Mean dose ratios for both TRUDOSE studies, stratified by underlying condition *.

Patient Group	Mean Dose Ratio Using Last Dose			Mean Dose Ratio Using Total Dose		
	TRUDOSE Pilot (*n* = 39)	TRUDOSE II (*n* = 44)	Pooled Data (*n* = 83)	TRUDOSE Pilot (*n* = 39)	TRUDOSE II (*n* = 44)	Pooled Data (*n* = 83)
Cervical dystonia	1.27	1.14	1.21 ^†^	1.15	1.10	1.12 ^†^
Blepharospasm	1.22	1.50	1.37 ^†^	1.20	1.34	1.28 ^†^
All patients	1.25	1.28	1.27 ^†^	1.17	1.19	1.18 ^†^

* Mean dose ratio is the ratio of incobotulinumtoxinA to onabotulinumtoxinA. ^†^ Statistically significant difference where the null hypothesis is dose ratio = 1, one-sample *t*-test with Bonferroni correction for multiple comparisons, *p* < 0.0001.

**Table 4:** Treatment characteristics for both TRUDOSE studies, stratified by underlying condition.

Characteristic	TRUDOSE Pilot (*n* = 39)		TRUDOSE II (*n* = 44)	
	OnabotulinumtoxinA	IncobotulinumtoxinA	OnabotulinumtoxinA	IncobotulinumtoxinA
**Cervical dystonia**				
Mean (SD) dosage per visit, U	127.3 (55.9)	144.5 (69.5)	188.6 (92.0)	194.9 (72.2)
Median (range) dosage, U	120.0 (41.4–257.1)	132.5 (42.5–366.0)	200 (70.9–487.1)	203.8 (81.8–376.7)
Mean (SD) total dose per patient per year, U/year	444.7 (347.2–542.2) *	536.3 (409.6–663.0) *^,†^	706.2 (374.9)	837.3 (336.7) ^†^
Inter-injection intervals, week	15.6	14.5	14.6	14.4
**Blepharospasm**				
Mean (SD) dosage per treatment, U	14.4 (12.2)	17.1 (13.8)	35.3 (23.1)	46.8 (31.4)
Median (range) dosage, U	11.2 (5.4–52.9)	12.0 (6.3–56.3)	40.0 (6.0–65.0)	46.0 (7.5–89.1)
Mean (SD) total dose per patient per year, U/year	50.4 (21.2–79.6) *	64.0 (33.3–94.7) *^,†^	133.2 (93.5)	207.2 (145.1) ^†^
Inter-injection intervals, week	16.3	14.2	16.2	13.9

* 95% confidence interval presented. ^†^ Statistically significant difference between the two botulinum toxins, paired *t*-test, *p* < 0.01. SD = standard deviation.

**Table 5:** Summary of adverse events across the pooled population.

	Pooled Population (*n* = 92)	
	Events, *n*	Patients, *n* (%)
**Any adverse event**	30	22 (23.9)
Serious adverse event	0	0
Severe adverse event	0	0
**Adverse events by treatment received**		
OnabotulinumtoxinA	15	12 (13.0)
IncobotulinumtoxinA	15	13 (14.1)
**Adverse events by underlying condition**		
Cervical dystonia (*n* = 58)	17	12 (20.7)
Blepharospasm (*n* = 34)	13	10 (29.4)

**Table 6:** Adverse events documented in >1 patient across the pooled population, stratified by the underlying condition.

Adverse Event, *n*	Pooled Population (*n* = 92)	
	OnabotulinumtoxinA	IncobotulinumtoxinA
**Cervical dystonia**		
Dysphagia	4	2
Head drop	2	2
Cervical extension weakness	1	1
Injection site pain	1	2
**Blepharospasm**		
Ptosis	3	4
Bruising	0	2

## Data Availability

Data are available upon reasonable request from Con Yiannikas at y.con@bigpond.com. These data can be requested by any qualified researchers who engage in rigorous, independent scientific research, and will be provided following review and approval of a research proposal and Statistical Analysis Plan (SAP) and execution of a Data Sharing Agreement (DSA). Data requests can be submitted at any time and the data will be accessible for 12 months, with possible extensions considered. For more information on the process, or to submit a request, visit the following link: https://www.abbvie.com/our-science/clinical-trials/clinical-trials-data-and-information-sharing/data-and-information-sharing-with-qualified-researchers.html (accessed on 13 July 2021).
